# Supplementary material for: Spectrally blue hydrated parent body of asteroid (162173) Ryugu
Source: Nat Commun. 2021 Oct 5;12:5837. doi: 10.1038/s41467-021-26071-8 (PMC8492871; doi:10.1038/s41467-021-26071-8)
Supplement: Supplementary file 1 — Supplementary Information [file 41467_2021_26071_MOESM1_ESM.pdf]

SUPPLEMENTARY INFORMATION

Spectrally blue hydrated parent body of asteroid (162173) Ryugu

Eri Tatsumi, Naoya Sakatani, Lucie Riu, Moe Matsuoka, Rie Honda, Tomokatsu Morota, Shingo Kameda, Tomoki Nakamura, Michael Zolensky, Rosario Brunetto, Takahiro Hiroi, Sho Sasaki, Sei'ichiro Watanabe, Satoshi Tanaka, Jun Takita, Cédric Pilorget, Julia de León, Marcel Popescu, Juan Luis Rizos García, Javier Licandro, Ernesto Palomba, Deborah Domingue, Faith Vilas, Humberto Campins, Yuichiro Cho, Kazuo Yoshioka, Hirotaka Sawada, Yasuhiro Yokota, Masahiko Hayakawa, Manabu Yamada, Toru Kouyama, Hidehiko Suzuki, Chikatoshi Honda, Kazunori Ogawa, Kohei Kitazato, Naru Hirata, Naoyuki Hirata, Yuichi Tsuda, Makoto Yoshikawa, Takanao Saiki, Fuyuto Terui, Satoru Nakazawa, Yuto Takei, Hiroshi Takeuchi, Yukio Yamamoto, Tatsuaki Okada, Yuri Shimaki, Kei Shirai, Seiji Sugita

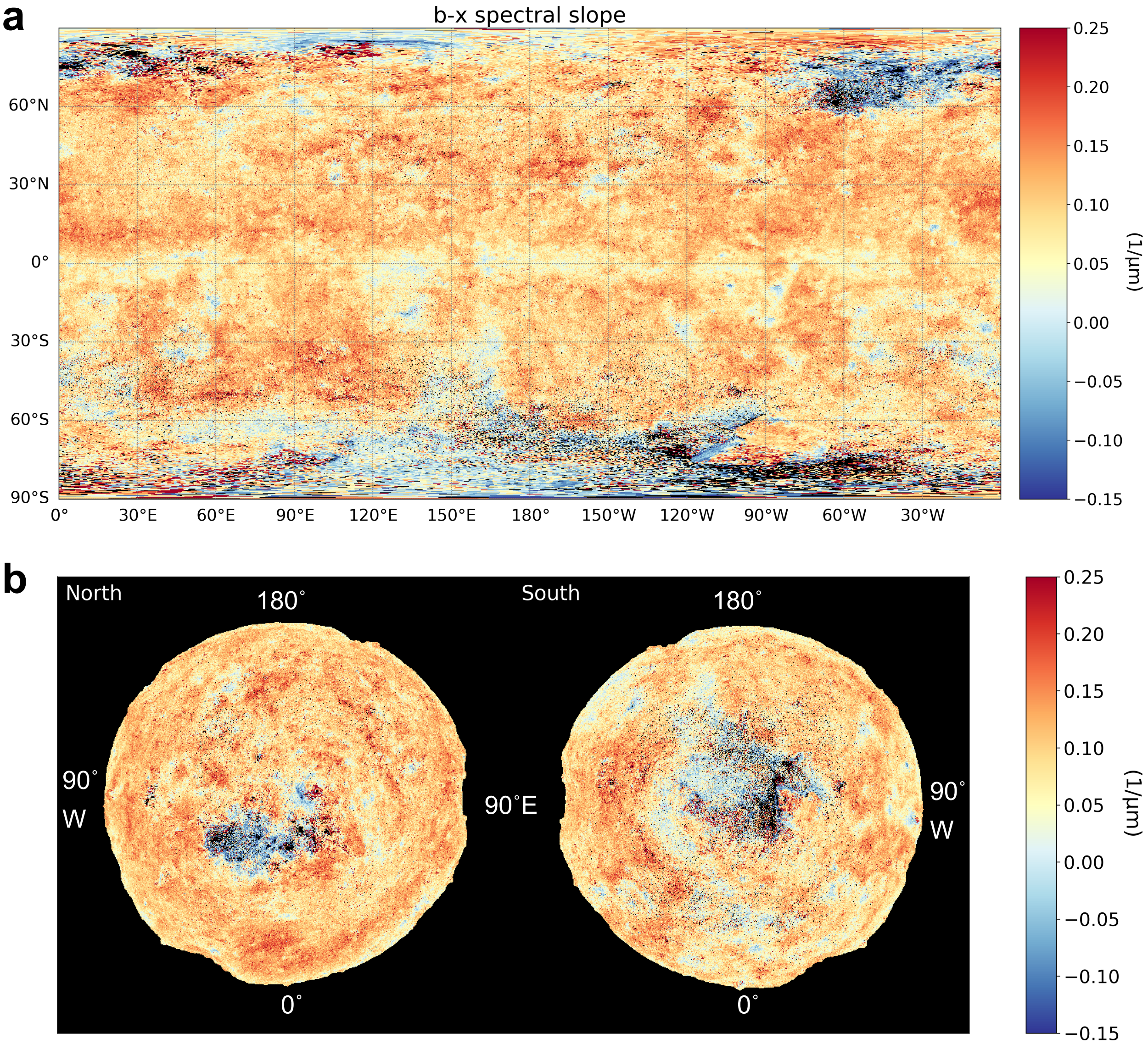

Supplementary Figure 1. Global spectral slope (b-x: 0.48 – 0.86 μm) distribution. The spectral slope [1/μm] between 0.48 to 0.86 μm was calculated by linear regression of normalized reflectance values at b (0.48 μm), w (0.70 μm), and x (0.86 μm) bands. **a** Cylindrical projection map. **b** Maps projected from the north and south poles.

Supplementary Table 1. ONC-T Image ids used for this study. All those images can be found in the archive page: https://data.darts.isas.jaxa.jp/pub/hayabusa2/paper/Tatsumi_2021/.

Item	Image id
Figures 1a, 6	hyb2_onc_20190228_130608_tvf – hyb2_onc_20190228_130744_tuf
Figure 1b	hyb2_onc_20191026_065643_tvf – hyb2_onc_20191026_065928_tuf
Figure 1c, 6	hyb2_onc_20190301_060828_tvf – hyb2_onc_20190301_061004_tuf
Figure 1d	hyb2_onc_20190301_115158_tvf – hyb2_onc_20190301_115334_tuf
Supplementary Figure 4a	hyb2_onc_20191036_065652_tvf
Supplementary Figure 4b	hyb2_onc_20190301_060828_tvf
Supplementary Figure 4c	hyb2_onc_20190301_115158_tvf
Supplementary Figure 4d	hyb2_onc_20190228_130608_tvf
Supplementary Movie 1	hyb2_onc_20190228_130608_tvf – hyb2_onc_20190228_181304_tuf
Supplementary Movie 2	hyb2_onc_20190301_060828_tvf – hyb2_onc_20190301_130955_tuf

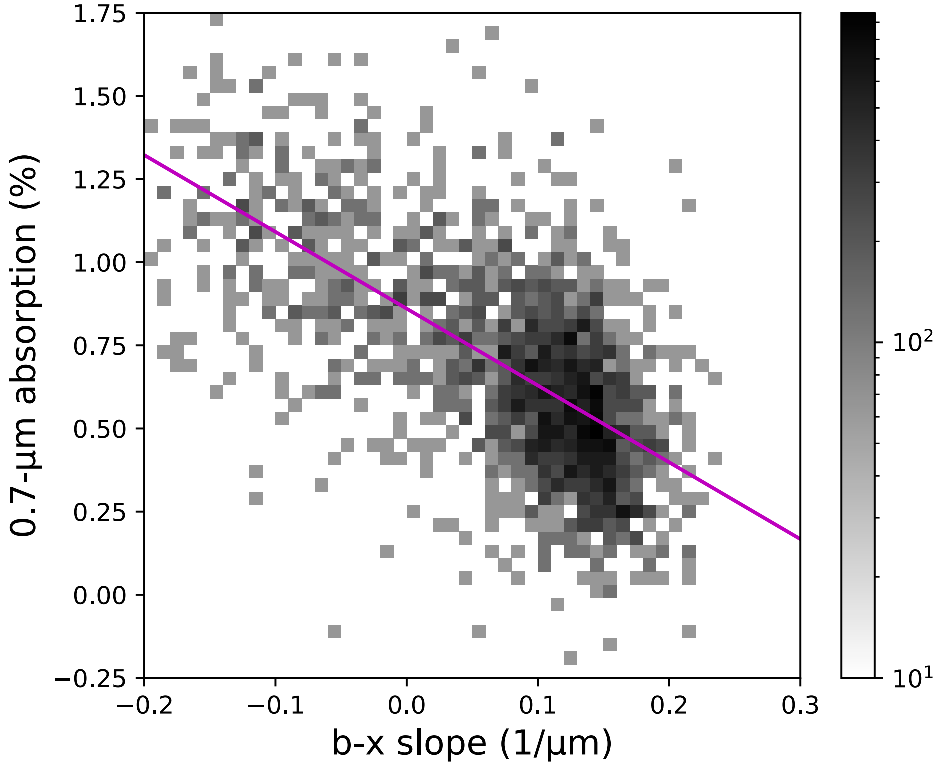

Supplementary Figure 2. Dependence of 0.7-µm band absorption to the b-x (480 – 860 nm) slope. Spectral slope and 0.7-µm band absorption at the north polar region, i.e., the data from central 394 x 394 pixels of Fig. 1a. Density plot indicates pixel numbers and the violet line indicates the linear regression (correlation coefficient -0.65). The color bar indicates the number of pixels in each bin.

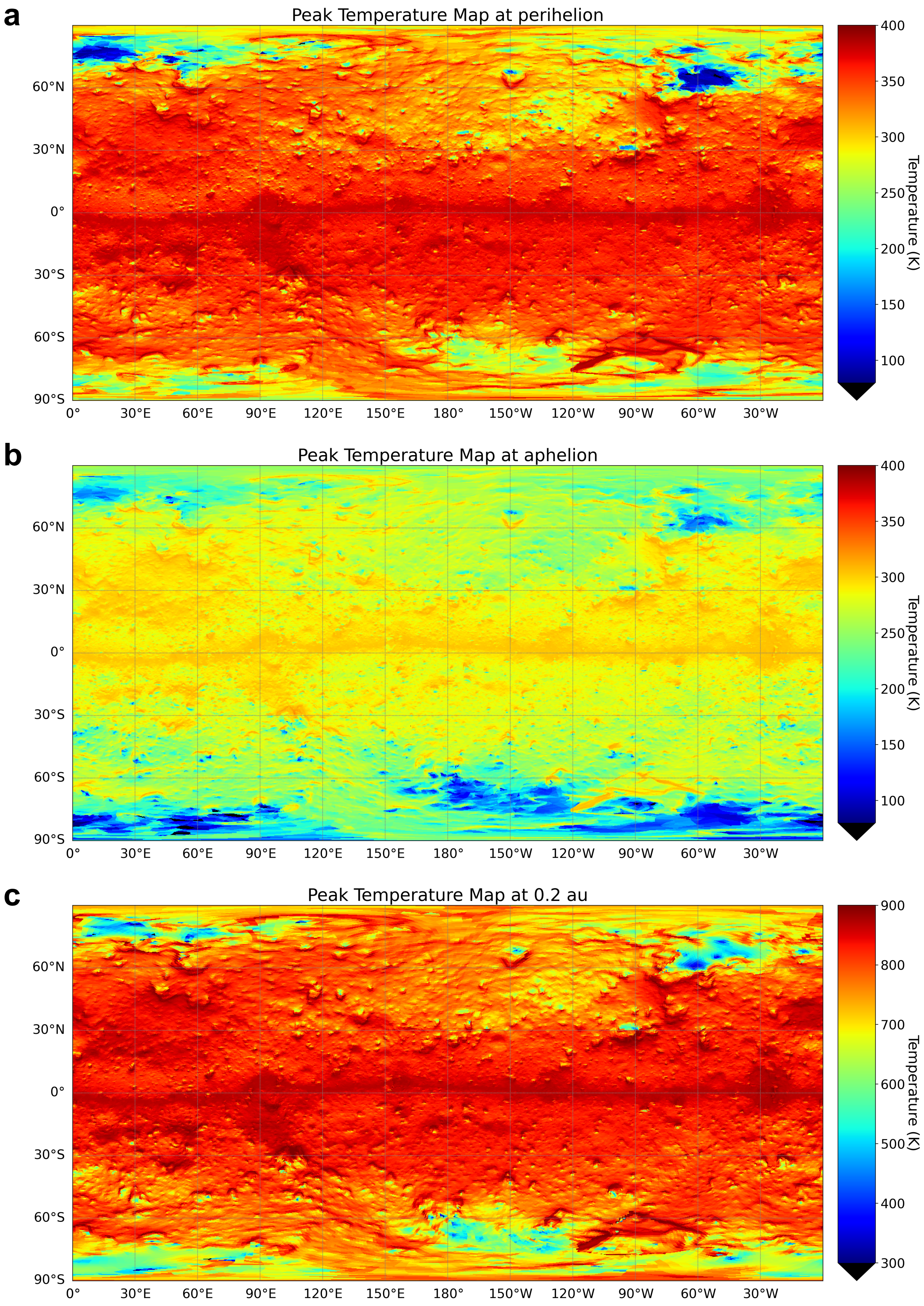
a
Peak Temperature Map at perihelion
60°N
30°N
0°
30°S
60°S
90°S
0°
30°E
60°E
90°E
120°E
150°E
180°
150°W
120°W
90°W
60°W
30°W
400
350
300
250
200
150
100
Temperature (K)
b
Peak Temperature Map at aphelion
60°N
30°N
0°
30°S
60°S
90°S
0°
30°E
60°E
90°E
120°E
150°E
180°
150°W
120°W
90°W
60°W
30°W
400
350
300
250
200
150
100
Temperature (K)
c
Peak Temperature Map at 0.2 au
60°N
30°N
0°
30°S
60°S
90°S
0°
30°E
60°E
90°E
120°E
150°E
180°
150°W
120°W
90°W
60°W
30°W
900
800
700
600
500
400
300
Temperature (K)

Supplementary Figure 3. **Simulated global peak temperature map.** Diurnal maximum surface temperature is calculated based on the shape model and the current pole orientation. **a** Temperature distribution at the current orbit at perihelion. **b** Temperature at the current orbit at aphelion. **c** Temperature distribution at the heliocentric distance of 0.2 au at equinox. Most of the surface exceeds 700 K where the phyllosilicates start to be decomposed, while some regions around poles are kept under 700 K. If Ryugu had experienced close encounter to the Sun which was hypothesized in [1], there could be a large difference in phyllosilicate amount between polar regions and the typical Ryugu surface. Nevertheless, such a large difference was not observed (See Fig. 3).

Supplementary Table 2. Summary of the NIRS3 dataset used for the characterization of the polar regions. The number of spectra indicates the number of NIRS3 spectra that fall into the region of interest and that were used to compute the average spectra displayed on Fig. 3. The spectra on the region of interest where extracted based on simultaneous observations of the ONC and NIRS3 instrument as explained in [2]. The comments describe some notes for observation, e.g., photometry correction [3] and Radiometric Calibration Coefficient (RCC) [4].

Observation	Region	Phase	Number of spectra	Comments
27th February 2019	Otohime A	After 1st Touchdown	23	No photometry correction
26th July 2019	North Pole	After 2nd Touchdown	75	Change of RCC
27th July 2019	Otohime B	After 2nd Touchdown	30	Change of RCC
26th October 2019	Otohime C	After 2nd Touchdown	100	Change of RCC

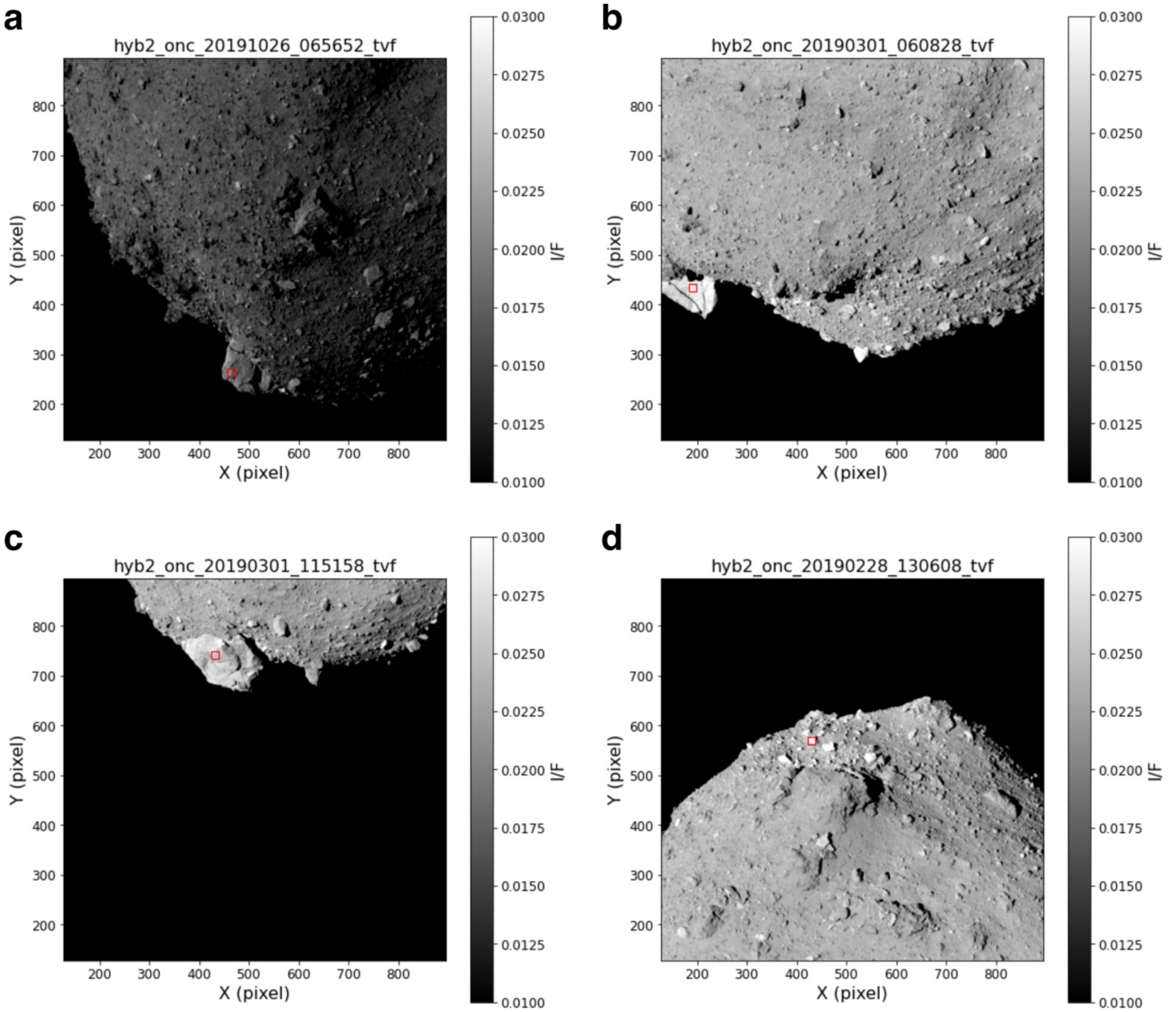

Supplementary Figure 4. Regions of interest for extracting spectra in Fig. 2. Each red square (15 pixels by 15 pixels) indicates the region of interest. **a** Otohime Facet A. **b** Otohime Facet B. **c** Otohime Facet C. **d** the north pole region. X and Y axes indicate pixel position in each image. The color bars indicate radiance factor (I/F) values (no unit). Image ids can be found in Supplementary Table 1.

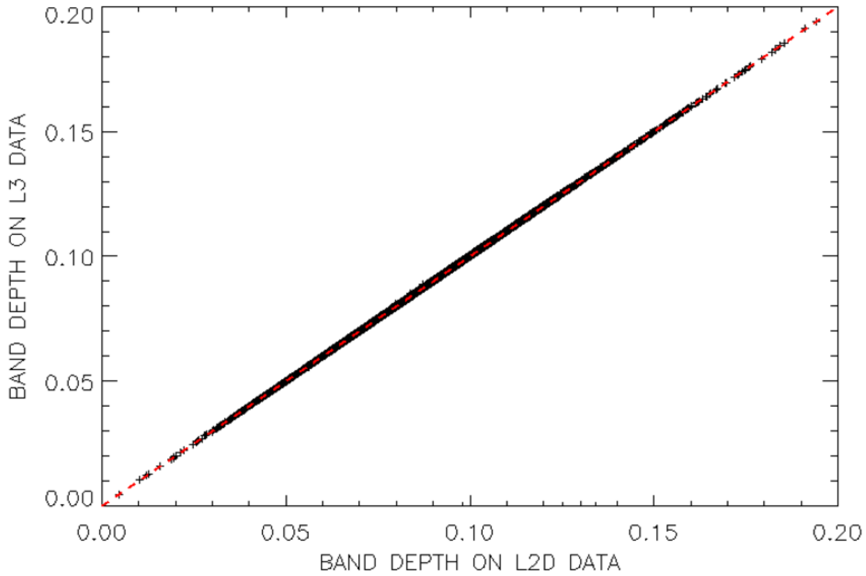

Supplementary Figure 5. Band depth measurement at 2.7 µm. Correlation of band depth at 2.72 µm measured from I/F data (L2D) and photometrically corrected data (L3), showing the band depth can be measured from I/F data without photometry correction. L2D data are available as the calibrated data on the Small Bodies Node of the NASA Planetary Data System (https://sbn.psi.edu/pds/resource/hayabusa2/nirs3.html). The photometry correction applied to L3 can be found in [3]. The red dashed line shows the line x=y.

Supplementary references

[1] Morota, T., et al. Sample collection from asteroid (162173) Ryugu by Hayabusa2: Implications for surface evolution. *Science* **368**, 654-659 (2020).

[2] Kitazato, K., et al. Thermally altered subsurface material of asteroid (162173) Ryugu. *Nature Astronomy* **5**, 246-250 (2021).

[3] Piloget, C., et al. Global-scale albedo and spectrophotometric properties of Ryugu from NIRS3/Hayabusa2, implications for the composition of Ryugu and representativity of the returned samples. *Icarus* **355**, 114126 (2021)

[4] Kitazato, K., et al. The surface composition of asteroid 162173 Ryugu from Hayabusa2 near-infrared spectroscopy. *Science* **364**, 272-275.
