# Supplementary material for: Spectrally blue hydrated parent body of asteroid (162173) Ryugu
Source: Nat Commun. 2021 Oct 5;12:5837. doi: 10.1038/s41467-021-26071-8 (PMC8492871; doi:10.1038/s41467-021-26071-8)
Supplement: Supplementary file 3 — Description of Additional Supplementary Files [file 41467_2021_26071_MOESM3_ESM.docx]

File Name: Supplementary Movie 1

Description: **Movie of North pole observations.** One rotational observations of North pole was conducted by the multi-band imager ONC-T. Top panels display the same values of Figures 1, the b-x (0.48 – 0.86 µm) spectral slope and 0.7-µm absorption depth. Bottom panels display the simulated values, the maximum temperature at perihelion (left) and the average photon dose (right).

File Name: Supplementary Movie 2

Description: **Movie of South pole observations.** One rotational observations of South pole was conducted by the multi-band imager ONC-T. Top panels display the same values of Figures 1, the b-x (0.48 – 0.86 µm) spectral slope and 0.7-µm absorption depth. Bottom panels display the simulated values, the maximum temperature at perihelion (left) and the average photon dose (right).
