# Supplementary figures and images for: Spectrally blue hydrated parent body of asteroid (162173) Ryugu
Source: Nat Commun. 2021 Oct 5;12:5837. doi: 10.1038/s41467-021-26071-8 (PMC8492871; doi:10.1038/s41467-021-26071-8)

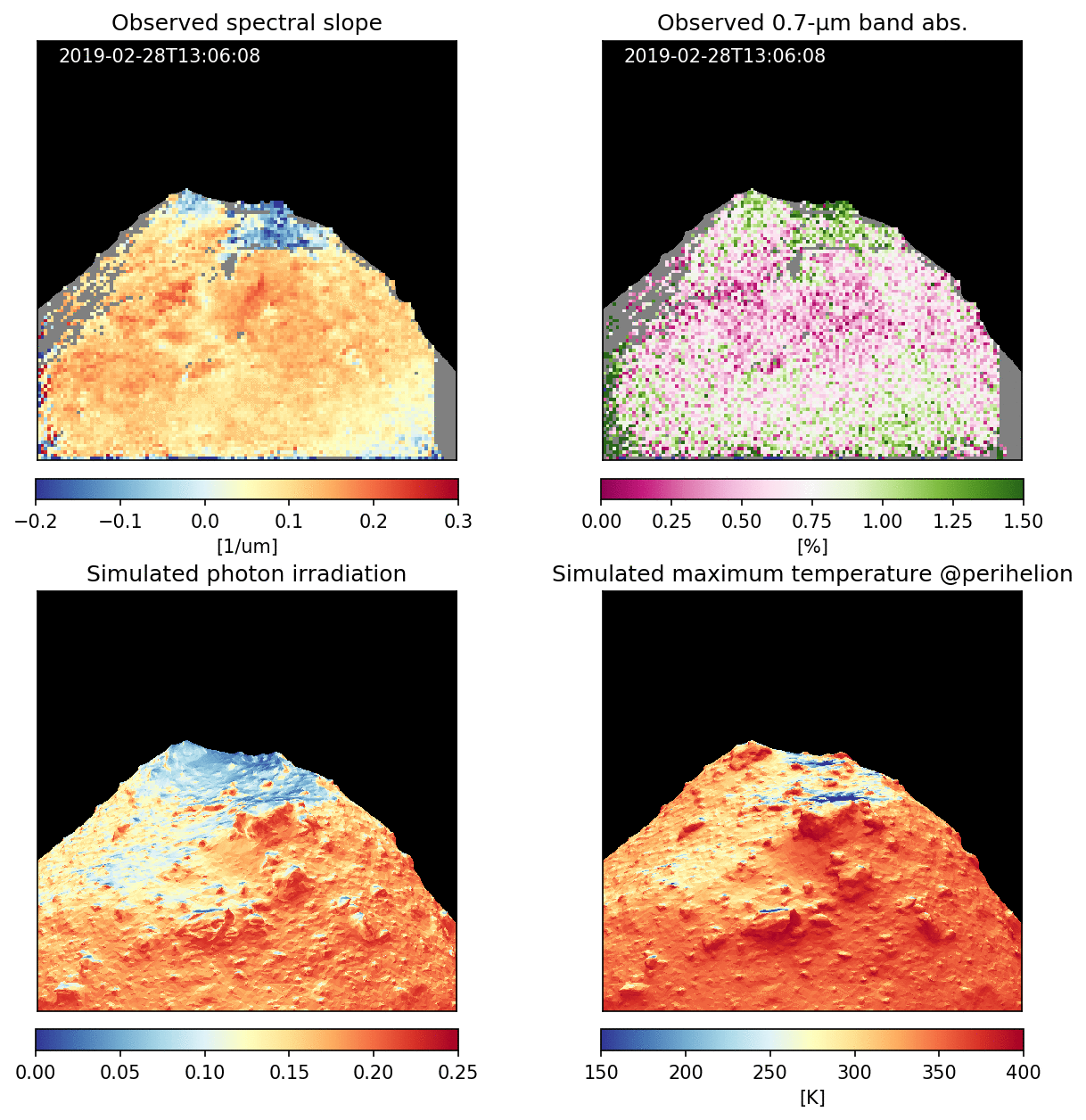

Supplement: Supplementary file 4 — Supplementary Movie 1 [file 41467_2021_26071_MOESM4_ESM.gif]

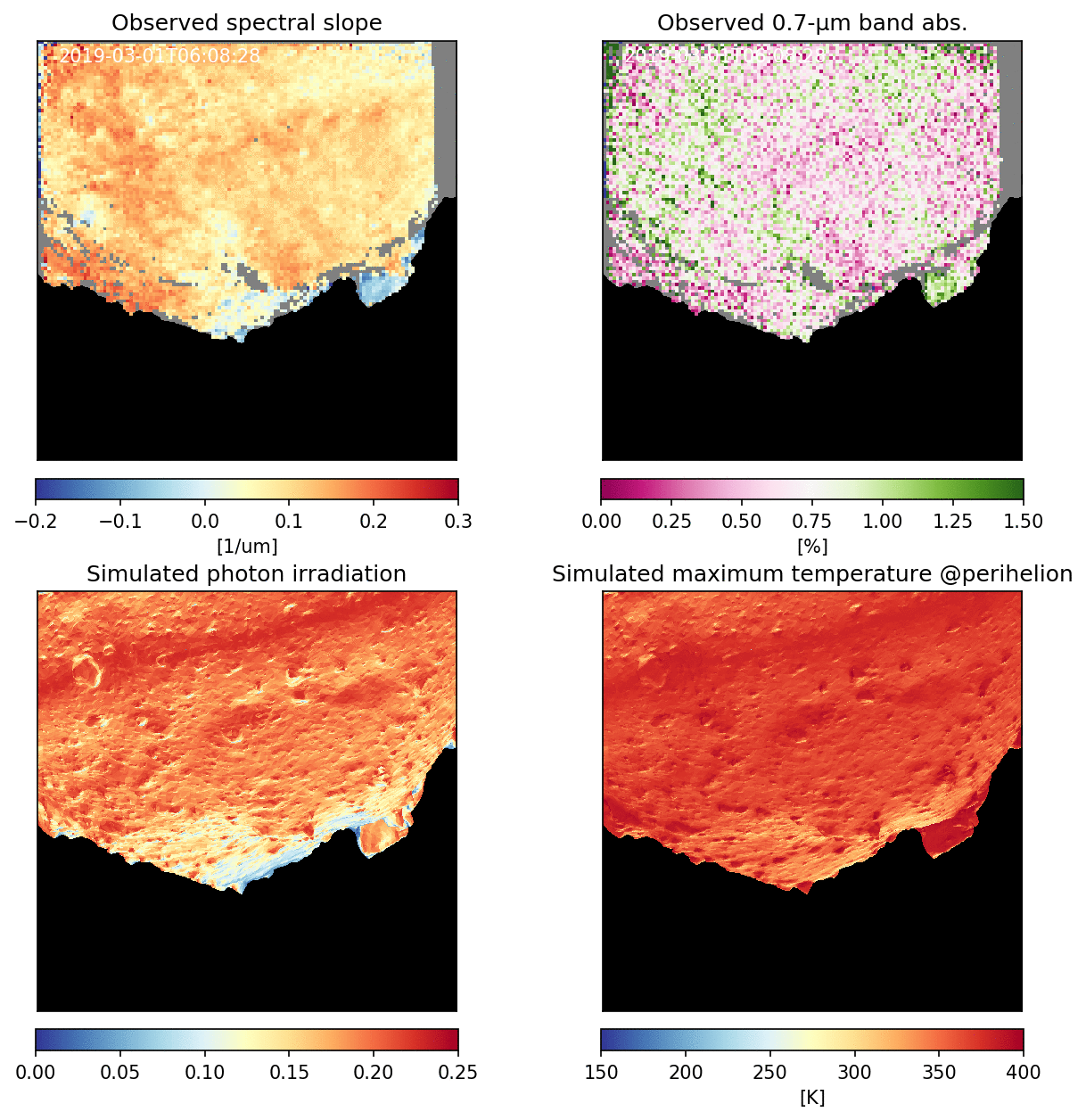

Supplement: Supplementary file 5 — Supplementary Movie 2 [file 41467_2021_26071_MOESM5_ESM.gif]
